# Hierarchical cluster analysis of labour market regulations and population health: a taxonomy of low- and middle-income countries

**DOI:** 10.1186/1471-2458-12-286

**Published:** 2012-04-18

**Authors:** Carles Muntaner, Haejoo Chung, Joan Benach, Edwin Ng

**Affiliations:** 1 Dalla Lana School of Public Health, University of Toronto, Toronto, Canada; 2 Bloomberg Faculty of Nursing, University of Toronto, Toronto, Canada; 3 Department of Healthcare Management, Korea University, Seoul, Republic of Korea; 4 Health Inequalities Research Group (GREDS), Employment Conditions Network (EMCONET), CIBER Epidemiología y Salud Pública (CIBERESP), Department of Experimental Sciences and Health, Pompeu Fabra University, Barcelona, Catalonia, Spain

## Abstract

**Background:**

An important contribution of the social determinants of health perspective has been to inquire about non-medical determinants of population health. Among these, labour market regulations are of vital significance. In this study, we investigate the labour market regulations among low- and middle-income countries (LMICs) and propose a labour market taxonomy to further understand population health in a global context.

**Methods:**

Using Gross National Product per capita, we classify 113 countries into either low-income (n = 71) or middle-income (n = 42) strata. Principal component analysis of three standardized indicators of labour market inequality and poverty is used to construct 2 factor scores. Factor score reliability is evaluated with Cronbach's alpha. Using these scores, we conduct a hierarchical cluster analysis to produce a labour market taxonomy, conduct zero-order correlations, and create box plots to test their associations with adult mortality, healthy life expectancy, infant mortality, maternal mortality, neonatal mortality, under-5 mortality, and years of life lost to communicable and non-communicable diseases. Labour market and health data are retrieved from the International Labour Organization's Key Indicators of Labour Markets and World Health Organization's Statistical Information System.

**Results:**

Six labour market clusters emerged: Residual (n = 16), Emerging (n = 16), Informal (n = 10), Post-Communist (n = 18), Less Successful Informal (n = 22), and Insecure (n = 31). Primary findings indicate: (i) labour market poverty and population health is correlated in both LMICs; (ii) association between labour market inequality and health indicators is significant only in low-income countries; (iii) Emerging (e.g., East Asian and Eastern European countries) and Insecure (e.g., sub-Saharan African nations) clusters are the most advantaged and disadvantaged, respectively, with the remaining clusters experiencing levels of population health consistent with their labour market characteristics.

**Conclusions:**

The labour market regulations of LMICs appear to be important social determinant of population health. This study demonstrates the heuristic value of understanding the labour markets of LMICs and their health effects using exploratory taxonomy approaches.

## Background

Labour markets and the regulations and institutions that shape them are emerging as important social determinants of health (SDOH) in a global context [1,2]. Increasingly, labour markets are reflected in global health discussions given the strong association between poor health and employment conditions such as unemployment, precarious work, informal work, temporary work, contract work, child labour, and slavery/bonded labour [1,3]. The current global economic crisis underscores the research need to consider how national and international labour markets operate and determine levels of population health [4].

In this article, we analyze data to understand the link between labour market regulations and population health among low- and middle income countries (LMICs). We create labour market taxonomies, or clusters, based on indicators of labour market inequality and poverty and empirically test the association between labor market clusters and population health. This current study builds upon our past work in two important ways [5]. Conceptually, we provide a deeper analysis of labour market regulations and welfare systems in LMICs, contributing to the growing yet sparse body of research on developing nations and population health [6]. Empirically, we conduct zero-order correlations to assess the strength of association between labour market factor scores and health outcomes, providing further insight on the differential impact of labour market regulations across clusters. In doing so, we demonstrate the heuristic value of conceptualizing labour markets as social determinants of population health in LMICs. Before proceeding to our analysis, we briefly review important labour market differences between high-income and LMICs, and resolve necessary theoretical and conceptual issues related to examining labour markets in LMICs.

### Literature review

#### Formal labour market institutions in high-income countries

Understanding the association between labour markets and population health involves discerning how global labour markets are heterogeneous, particularly between high-income and LMICs. In broad terms, labour markets are comprised of two sectors: formal and informal [7]. High-income countries are often characterized by formal sectors where workers are protected and supported by a range of regulations, programs, and arrangements collectively referred to as labour market institutions. Labour institutions primarily consist of five components: hiring, deployment, termination, post-termination, and union density/collective bargaining [8]. The first three components are often considered part of employment protection, referring to both "regulations concerning hiring (e.g., rules favouring disadvantaged groups, conditions for using temporary or fixed-term contracts, training requirements) and firing (e.g., redundancy procedures, mandated prenotification periods and severance payments, special requirements for collective dismissals and short-time work schemes) [9] p. 50." Post-termination programs are not forms of employment protection but instead provide supports (e.g., income support, retraining, and job search) to workers who are in between jobs. Unions and collective bargaining agreements defend and amend the protection of workers and are used to set wages [10,11].

Historically, the strictness of regulation, the generosity of support programs, and the extent of collective bargaining tends to reflect workers' participation with the development of collective labour rights, the labour movement, and the policies and labour market developed by welfare state regimes. Labour institutions are closely related to the virility of the welfare state [11], are mediators in how the state regulates the labour market (e.g., provisions for collective bargaining), and are strongly associated with welfare state regime type in wealthy countries [12-14]. According to Esping-Andersen [15,16], three welfare state regimes emerged after World War II among high-income countries: (i) Conservative-Corporatist, (ii) Liberal, and (iii) Social Democratic. Each regime is based on historical power dynamics between organized labour, government, and business associations and degrees of de-commodification, which occurs when services are rendered as a matter of right, and when persons can maintain a livelihood without reliance on the market [15]. Types of welfare states range from high government intervention, generous welfare systems found in Social Democratic countries (e.g., Sweden) to residual, means-tested systems in Liberal nations (e.g., US) to Conservative regimes (e.g., Germany), who fall in the middle with moderate levels of service provision and citizen supports. Welfare state regime theory has received its share critiques ranging from theoretical (e.g., gender-blind concept of decommodification) and methodological (e.g., the use of one standard deviation around the mean to classify the countries into three regimes) and empirical (e.g., inability for original results to be replicated) limitations [17,18].

Labour market institutions and welfare states have been recently incorporated into public health research and widely studied in high-income, OECD countries [13,19-26]. Recent studies suggest that formal labour markets with high union density and collective bargaining coverage [2,27,28] and greater investments in active labour market policies (Holland et al. 2011) are both strong predictors of better population health. Other work reveals that labour market institutions, measured with OECD's employment protection legislation index (EPL-index), are positively associated with health outcomes [5]. Labour market clusters based on EPL-indices mirror Esping-Andersen's welfare regime typology and substantiates the link between egalitarian institutions and better population outcomes with Social Democratic clusters (e.g., Belgium, Denmark, Finland, Italy, Norway, Sweden) having the most advantaged health outcomes, followed in order by Corporatist-Conservative clusters (e.g., Austria, France, Germany, Greece, Japan, Netherlands, Portugal, Spain) and Liberal clusters (e.g., Australia, Canada, Ireland, New Zealand, Switzerland, UK, US) [5]. To date, the relation between labour market institutions and population health in high-income countries appears to be mediated by SDOH-based policies and labour market interventions that characterize distinct welfare state regime types [24]. These SDOH-based policies and interventions include but are not limited to job security, minimum wage and hours of work legislation, reach of safety and health legislation into the workplace, workers compensation systems, public health infrastructure, social protection including pensions, and state investment in social services [2].

#### From labour market institutions to regulations in low- and middle-income countries

Whereas formal sectors consist of labour market institutions with a welfare mix of market, state, and family arrangements, the labour markets of LMICs largely operate within the informal sector (e.g., activities and income that are partially or fully outside of government regulation and taxation) [2]. We define the informal economy as "not an individual condition but a process of income generation characterized by one central feature: it is unregulated by the institutions of society, in a legal and social environment in which similar activities are regulated [29] p. 12." As a result, this sector tends to lack statutory regulation to protect working conditions, wages, occupational health and safety, and injury insurance [2,7]. Informal labour markets emerge in the absence of state regulation and labour market institutions [30]. Recent estimates on the size of the informal sector in LMICs, excluding its share of non-agricultural employment, range between one-fifth and four-fifths [31]. In terms of its contribution to GDP, the informal sector accounts for between 25% and 40% of annual output in LMICs in Asia and Africa [31]. Compared to high-income countries, informal economies have fewer workers engaged in post-industrial financial capitalism and significantly more working in agriculture and in the production of primary goods [32]. Interestingly, the characteristics of informal sectors *today *resembles where high-income countries *were *at the turn of the 20th century [2,33-35], or put differently, what formal sectors in high-come countries provide through strong labour market institutions (e.g., job security, minimum wage, safety legislation into the workplaces) reflects what informal sectors in LMICs fail to provide through weak regulations, resulting in precariousness, poverty wages, and hazardous working environments.

To comprehensively understand the labour markets and welfare contexts of LMICs, we turn to the theoretical and empirical work of Gough and Wood [36-38], who modify and apply Esping-Andersen's [15,16] concept of welfare state regimes to regions and countries with problematic states and imperfect labor markets. In their view, LMICs can be characterized as either *informal security regimes *or *insecurity regimes *[36]. The former describes institutional arrangements where people rely heavily upon community and family relationships to meet their security needs to various degrees, while the latter refers to arrangements which generate gross insecurity and block the emergence of stable informal labour market arrangements to mitigate these insecurities. These two regimes contribute to a broader, comparative theory comprised of four components: *institutional conditions *(e.g., the pervasiveness and character of labour markets, the legitimacy of the state, cultural values and the position of the country in the global system), *institutional responsibility-matrix *(e.g., institutional landscape within which people have to pursue their livelihoods and well-being objectives, referring to the role of government, community, private sector market activity, and the household in mitigating insecurity and well-being), *systems of stratification and mobilization *(e.g., the existing distribution of power in a society and the range of societal inequalities), and *welfare outcomes *(e.g., the extent of poverty and other measures of low or inadequate resources) [38].

For our discussion, the regulatory character of informal labour markets within *institutional conditions *is especially important because labour market characteristics such as labour market inequality and poverty are pervasive within and between LMICs. Generally, as labour market inequality and poverty decrease, state intervention erodes the de facto authority of informal markets, replacing informal regulations with formal institutions [7]. However, this process is problematic given the labour market burdens and barriers facing LMICs. First, earnings levels are diminutive and poverty is widespread despite long work hours [2,7]. Wages in LMICs are often no more than two U.S. dollars per day and workers often have irregular and long hours [39]. Second, women are more deprived in LMICs --their earnings are lower and their work is more likely to be informal and in causal positions compared to their high-income counterparts [7]. Third, workers earn low incomes and receive irregular and unpredictable income [39]. As such, unregulated low wages may force workers to sell their labour for less than subsistence income [29]. Forth, the lack of regular wage employment opportunities often leads individuals to create their own self-employment opportunities [40]. Fifth, poverty among those who work is a bigger problem than unemployment. In 2009, the numbers of those unemployed versus working poor were approximately 200 million versus 1.3 billion people, respectively [31]. Sixth, informal sectors in LIMICs results in state revenue losses by reducing taxes and social security contributions paid and therefore the availability of funds to support SDOH-based policies and programs. Seventh, the dire working conditions in informal sectors such as child labour, slave labour, and lower-than-subsistence wage levels, are exacerbated in informal sectors [41]. The insufficient wages of parents often forces children to venture into the labour market at a very young age [29]. Given these stark differences between formal and informal sectors [14,36], theoretical and conceptual adjustments are necessary to adequately characterize the labour markets in LMICs [5,14]. One option is using more direct measures of labour market experiences and outcomes such as levels of inequality and poverty.

Our brief review here finds support that labour market institutions are important determinants of population health in wealthy countries; however, more research and conceptual adjustments are needed to advance similar work among LMICs. Our study aims to fill this void, both theoretically and empirically, by dividing countries into low-income and middle-income groups, and generating a taxonomy of labour market clusters using inequality and poverty indicators within each income group. Our research questions are three-hold: (1) How do LMICs cluster together across labour market regulations, measured with inequality and poverty indicators? (2) What is the strength of association between labour market regulations and population health? (3) Are more egalitarian labour market clusters associated with better population health outcomes?

## Methods

### Data sources and variables

Labour market variables are retrieved from the International Labour Organization's (ILO) Key Indicators of Labour Markets (KILM) database [42]. We conceptualize labour market regulations in LMICs as two factor scores, composed of three variables measured twice each. The first factor score measures inequality in the labour market with three standardized variables: estimated earned income ratio between male and female workers (1999, 2003); labour force participation gap between female and male workers (1997, 2003); and employment to population ratio (1997, 2003). The second factor score quantifies poverty and income level in labour market, also using three standardized variables measured twice: percentage of children in labour market (1997, 2003); percentage of workers that are poor (1997, 2003); and average income level (1999, 2003).

Health outcomes are downloaded from the World Health Organization's Statistical Information System (WHOSIS) [43]: female and male adult mortality rate, 2004 (probability of dying per 1,000 population between 15 and 60 years); female and male healthy life expectancy at birth, 2002 (HALE) (years); infant mortality rate, 2004 (per 1,000 live births); maternal mortality ratio, 2000 (per 100,000 live births); neonatal mortality rate, 2000 (per 1,000 live births); under-5 mortality rate, 2004 (per 1,000 live births); years of life lost to communicable diseases, 2002 (%); and years of life lost to non-communicable diseases, 2002 (%). Our rationale for using these outcomes is three-fold. First, the health outcomes are well-structured data, complete, and available among LMICs. Second, the WHOSIS is a central source of metadata of health-related indicators and is widely used by WHO, the World Bank, and other international organizations. Third, these health outcomes are highly sensitive to social determinants, including labour market characteristics, and are commonly used in comparative population health research [1].

## Statistical analyses

### Low- and middle-income classification

For the classification of countries based on national income, we use Gross National Product per capita (GNPpc) of year 2000, generated through the World Bank's Atlas Method (adjusted by exchange rate). Two clear groups of countries emerge with a cut-point in log10 of GNPpc [44]: low-income (n = 71) and middle-income (n = 42).

### Cluster analyses and construction of factor scores

For LMICs, we construct two factor scores based on labour market poverty and labour market inequality indicators. Factor analyses using principal component analysis (PCA) are conducted, and the reliability of factor scores are evaluated with Cronbach's alpha. Using the regression method, all indicators used to construct factor scores showed high factor loadings, with Cronbach's alpha scores - 0.934 and 0.913 for labour market inequality and labour market poverty, respectively. Using these two labour market factor scores, we conduct a series of hierarchical cluster analyses to generate clusters of countries using Ward's method of measuring squared Euclidean distance. Because no single indicator adequately measures labour markets in LMICs, we apply PCA to reduce the number of inequality and poverty variables and to obtain two factors containing the greatest variance [45]. PCA also accommodates our interval-level data and does not assume our data satisfies a specific statistical model [45]. We use hierarchical clustering methods with Ward's linkage over other techniques such as partition-clustering with k-means for pragmatic reasons. First, hierarchical clustering builds a binary tree (e.g., dendrogram) that successively merges similar LMICs, which allows us to rank clusters based on labour market factor scores and visually interpret the resulting algorithm [46]. Second, Ward's linkage performs well with our data set given our clusters are similar in sample sizes and number of observations [46]. Third, partition-clustering with k-means requires pre-specifying the number of clusters, *k*, to create distinct non-overlapping groups [47]. Since we had no preconceived notion on how many labour market clusters would emerge, we deemed this method unsuitable for our needs. We analyze the bivariate associations between labour market factors scores, inequality and poverty, using zero-order correlations. Box plots are also created to show population health distributions within and between labour market clusters. We use box plots over ANOVA techniques because the latter is vulnerable to the presence of outliers and are based on statistical assumptions (e.g., normal distributions and equal variance), which our small cluster sizes fail to meet [48]. All analyses are conducted using Stata version 10.0 [49].

## Results

Originally our dataset included a total of 172 LMICs (61 middle-income and 111 low-income countries). Due to missing data-points, 59 countries are excluded. A final sample of 113 countries (42 middle-income and 71 low-income countries) are retained and categorized into labour market clusters.

### Cluster of labour markets by national income

Table 1 presents our sample of countries categorized into low- and middle-income groups and clustered into six labour market groups based on inequality and poverty factor scores. Regional distributions of clusters show that middle-income countries are mostly East Asia and Eastern Europe, and Latin America, with a couple of African countries while low-income countries are predominantly African, South East Asian, and Caribbean nations.

**Table 1:** Taxonomy of labour market clusters by national income

	More Equal	← Labour Market →	Less Equal
**Middle-Income**	**Residual**	**Emerging**	**Informal**
	
	The Bahamas, Croatia, Czech Rep, Hong Kong, Hungary, Jamaica, Korea Rep, Latvia, Lithuania, Poland, Russian Fed, Singapore, Slovak Rep, Slovenia, Thailand, Uruguay	Argentina, Brazil, Chile, Colombia, Costa Rica, Ecuador, Fiji, Kuwait, Malaysia, Mexico, Panama, Paraguay, Peru, South Africa, Trinidad and Tobago, Venezuela	Bahrain, Belize, Botswana, El Salvador, Lebanon, Oman, Saudi Arabia, Tunisia, Turkey

Low-Income	Post-Communist	Less Successful Informal	Insecure
	
	Albania, Armenia, Belarus, Bolivia, Bulgaria, Cambodia, China, Ghana, Indonesia, Moldova, Mongolia, Papua New Guinea, Philippines, Romania, Tajikistan, Ukraine, Uzbekistan, Viet Nam	Algeria, Cape Verde, Cote d'Ivoire, Dominican Rep, Egypt, Equatorial Guinea, Guatemala, Guyana, Honduras, India, Iran, Jordan, Mauritania, Morocco, Nicaragua, Nigeria, Pakistan, Sri Lanka, Sudan, Swaziland, Syrian Arab Rep, Yemen Rep	Bangladesh, Benin, Burkina Faso, Burundi, Cameroon, Central African Rep, Chad, Comoros, Congo Dem Rep, Congo Rep, Eritrea, Ethiopia, Gambia, Guinea-Bissau, Haiti, Kenya, Lao PDR, Madagascar, Malawi, Mali, Mozambique, Namibia, Nepal, Niger, Rwanda, Senegal, Tanzania, Togo, Uganda, Zambia, Zimbabwe

#### Middle-income countries

Among middle-income countries, three labour market clusters emerged. Cluster 1 represents mostly East Asian and Eastern European countries with a handful of Caribbean nations (n = 16; The Bahamas, Croatia, Czech Rep, Hong Kong, Hungary, Jamaica, Korea Rep, Latvia, Lithuania, Poland, Russian Fed, Singapore, Slovak Rep, Slovenia, Thailand, and Uruguay). These regions are marked by an emphasis on industrialization and thus incorporation of rural workers into urban industrial centers [50,51]. Mass growth in urban working populations necessitated the development of labour contracts. The relationship among workers, companies, and governments are partially democratic, embedded in strong labour regulations, and overseen by authoritarian regimes. For these reasons we name this cluster "Residual."

Cluster 2 includes mostly Central and South American countries, South Africa, and Kuwait (n = 16; Argentina, Brazil, Chile, Colombia, Costa Rica, Ecuador, Fiji, Kuwait, Malaysia, Mexico, Panama, Paraguay, Peru, South Africa, Trinidad and Tobago, Venezuela). These countries have undergone limited industrialization with stagnated periods of economic development and have not incorporated as many rural populations as countries representing Cluster 1, Residual Labour Markets [50,51]. Nevertheless, the employment opportunities in urban centers have attracted immigrants from rural areas and adjacent countries into cities, producing massive urban slums and large informal sectors. Accordingly, we label this labour market cluster as "Emerging."

The third and last cluster among middle-income countries includes economies with lagged industrialization due to civil wars and other crises (n = 10; Bahrain, Belize, Botswana, El Salvador, Lebanon, Oman, Saudi Arabia, Tunisia, Turkey). For these countries, the majority of national income is derived from oil exports and authoritarian rule of law. Given that these labour markets are predominantly informal, consisting of contract work, we label this cluster "Informal."

#### Low-income countries

Low-income countries also clustered into three groups. The first cluster identifies former communist countries (n = 18; Albania, Armenia, Belarus, Bolivia, Bulgaria, Cambodia, China, Ghana, Indonesia, Moldova, Mongolia, Papua New Guinea, Philippines, Romania, Tajikistan, Ukraine, Uzbekistan, Viet Nam). The developmentalist and universalistic tendencies [50,52] of former communist countries have enabled them to distinguish themselves from the rest of low-income countries through industrialization and relatively lower levels of poverty. We label this labour market cluster as "Post-Communist."

Cluster 2 represents a geographically diverse group of low-income nations (n = 22; Algeria, Cape Verde, Cote d'Ivoire, Dominican Rep, Egypt, Equatorial Guinea, Guatemala, Guyana, Honduras, India, Iran, Jordan, Mauritania, Morocco, Nicaragua, Nigeria, Pakistan, Sri Lanka, Sudan, Swaziland, Syrian Arab Rep, Yemen Rep). These countries are not as homogenous as "Post-Communist" labour markets. We name this cluster "Less Successful Informal", following the informal labour market terminology used in middle-income countries.

Cluster 3 is the largest group of low-income countries and includes the world's poorest nations (n = 31; Bangladesh, Benin, Burkina Faso, Burundi, Cameroon, Central African Rep, Chad, Comoros, Congo Dem Rep, Congo Rep, Eritrea, Ethiopia, Gambia, Guinea-Bissau, Haiti, Kenya, Lao PDR, Madagascar, Malawi, Mali, Mozambique, Namibia, Nepal, Niger, Rwanda, Senegal, Tanzania, Togo, Uganda, Zambia, Zimbabwe). These countries exhibit higher levels of labour market poverty compared to other clusters, and have long histories of wars, natural disasters, and epidemics which undermine the functioning of the nation state. We designate this cluster as "Insecure."

### Descriptive summaries of labour market factor scores

Table 2 presents the mean values of labour market factor scores by income group and labour clusters. Lower 'labour market inequality factor scores' indicate greater levels of labour market equality while higher 'labour market poverty factor scores' indicate greater levels of poverty. Across income-groups, middle-income countries are advantaged with higher levels of labour market equality (*M *= -0.36, *SD *= 0.83) and lower levels of poverty (*M *= -0.42, *SD *= 0.45) compared to income-income nations (inequality, *M *= 0.08, *SD *= 1.12; poverty, *M *= -0.42, *SD *= 0.45). Inequality and poverty factor scores are significantly correlated with each other in low-income countries, but not in middle-income countries (data not shown). Comparisons between labour market clusters across poverty factor scores revealed that Residual markets (*M *= -0.67, *SD *= 0.25) had the lowest poverty scores, followed by Emerging (*M *= -0.34, *SD *= 0.28), Informal (*M *= -0.27, *SD *= 0.62), Post-Communist (*M *= -0.02, *SD *= 0.40), Less Successful Informal (*M *= -0.18, *SD *= 0.58), and Insecure (*M *= 1.49, *SD *= 0.43) clusters. The factor scores for labour market inequality did not follow this pattern; instead clusters ranked from the most advantaged to the least were as follows: Less Successful Informal (*M *= -1.32, *SD *= 0.41), Informal (*M *= -1.29, *SD *= 0.85), Emerging (*M *= -0.58, *SD *= 0.27), Residual (*M *= 0.45, *SD *= 0.32), Post-Communist (*M *= 0.65, *SD *= 0.59), Insecure (*M *= 0.73, *SD *= 0.72).

**Table 2:** Descriptive statistics of labour market factor scores by national income and labour market clusters

Clusters Variables	Middle-Income				Low-Income			
	
	Residual (n = 16)	Emerging (n = 16)	Informal (n = 10)	Middle-income Total (N Range = 43-52)	Post-Communist (n = 18)	Less Successful Informal (n = 22)	Insecure (n = 31)	Low-Income Total (N Range = 70-92)
	
	M (SD)	M (SD)	M (SD)	M (SD)	M (SD)	M (SD)	M (SD)	M (SD)
Labour market inequality factor score (std.)^a^	0.45 (0.32)	-0.58 (0.27)	-1.29 (0.85)	-0.36 (0.83)	0.65 (0.59)	-1.32 (0.41)	0.73 (0.72)	0.08 (1.12)

Labour market poverty factor score (std.)^b^	-0.67 (0.25)	-0.34 (0.28)	-0.27 (0.62)	-0.42 (0.45)	-0.02 (0.40)	0.18 (0.58)	1.49 (0.43)	0.70 (0.85)

M, Mean; SD, Standard Deviation

^a^Lower 'Labour market inequality factor scores' indicate greater levels of labour market equality

^b^Higher 'Labour market poverty factor scores' indicate greater levels of labour market poverty

### Association between factor scores and health indicators

Table 3 presents zero-order correlations coefficients between labour market inequality and poverty factor scores and population health across LMICs. In both middle-income and low-income countries, labour market poverty was significantly and negatively associated with HALE (both sexes), years of life lost to non-communicable diseases, and positively with maternal mortality rate, infant mortality rate, under 5-year mortality rate, neonatal mortality, adult mortality (both sexes), and years of life lost to communicable diseases. Overall, there was a strong correlation between labour market poverty and population health in LMICs --decreases in poverty were associated with improvements for each health outcome.

**Table 3:** Zero-Order correlation coefficients between labour market factor scores and health outcomes

Factor Scores Health Outcomes	Middle-Income Countries		Low-Income Countries	
	
	Labour Market Inequality Factor Score	Labour Market Poverty Factor Score	Labour Market Inequality Factor Score	Labour Market Poverty Factor Score
Healthy life expectancy at birth - Male	-0.0177	-0.6033*	-0.4021*	-0.7350*

Healthy life expectancy at birth - Female	0.1543	-0.5716*	-0.3791*	-0.7637*

Maternal mortality	-0.2263	0.5474*	0.4549*	0.7830*

Infant mortality rate	-0.1668	0.7184*	0.3302*	0.7498*

Under 5-year mortality rate	-0.1593	0.6945*	0.3638*	0.7754*

Neonatal mortality	-0.2172	0.7411*	0.1890*	0.6161*

Adult Mortality - Male	0.1997	0.4427*	0.3858*	0.6060*

Adult Mortality - Female	0.0255	0.4807*	0.3468*	0.6499*

Years of life lost to communicable diseases	-0.1654	0.5949*	0.2589*	0.7962*

Years of life lost to non-communicable diseases	0.2394	-0.5920*	-0.2170	-0.7846*

*p-value < 0.05

Correlations between labour market inequality and population health show a similar pattern among low-income countries, and are significantly correlated with same health outcomes in the same direction with the exception of years of life lost to communicable diseases. Increases in labour market equality in low-income countries correspond with accompanying increases in population health. The relationship between labour market inequality and population health among middle-income countries; however, were all non-significant.

### Labour market clusters and population health

**Figure 1:**
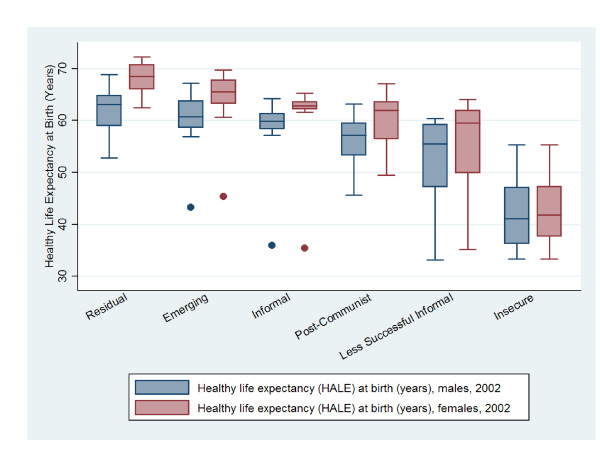
**Male and female healthy life expectancy (HALE) in years by labour market clusters**.

**Figure 2:**
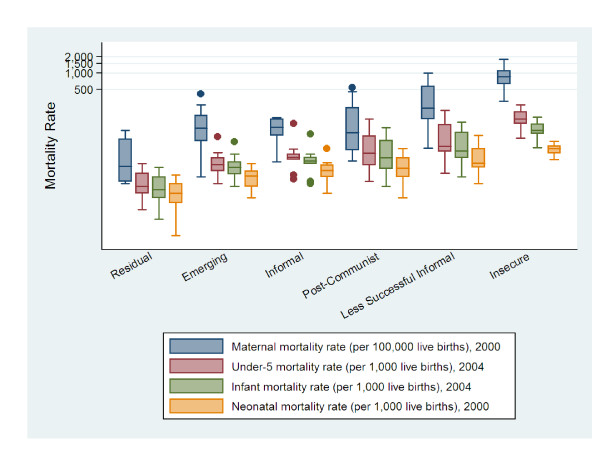
**Maternal and child health indicators by labour market clusters**.

**Figure 3:**
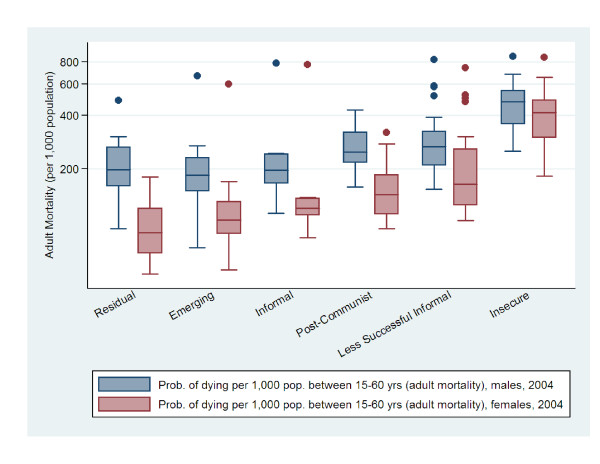
**Male and female adult mortality rate by labour market clusters**.

**Figure 4:**
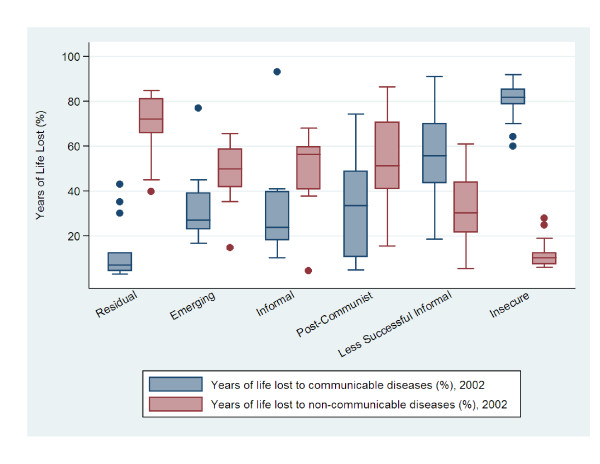
**Years of life lost to communicable and non-communicable diseases by labour market clusters**.

To compare labour market clusters and the distribution of health outcomes, Figures 1, 2, 3, and 4 present box^1 ^plots on HALE (both sexes), maternal and child health indicators, adult mortality (both sexes), and years of life lost to communicable and non-communicable diseases, respectively. The juxtaposition of box plots for HALE (both sexes), maternal and child health indicators, adult mortality (both sexes), and years of life lost to communicable diseases show clear separation between labour market clusters. Distributions indicate that more egalitarian clusters exhibit better health outcomes compared to their cluster counterparts. Furthermore, health distributions are consistently graded across labour market clusters, from best to worst, beginning with Residual markets, followed by Emerging, Informal, Post-Communist, Less Successful Informal, and Insecure. The outstanding exception is years of life lost to non-communicable diseases (Figure 4), which shows a positive relationship with labour market equality. Increases in labour market equality are associated with increases in lost years due to non-communicable diseases, supporting the finding that as countries make progress in developing economically and reducing labour market levels of inequality and poverty, the burden of diseases shifts from communicable (e.g., lower respiratory infections, HIV/AIDS, diarrheal diseases) to non-infectious diseases (e.g., heart disease and cancer) [53-55].

## Discussion

We find strong support for the idea that labour market clusters in LMICs are associated with adult mortality, healthy life expectancy, infant mortality, maternal mortality, neonatal mortality, under-5 mortality, and years of life lost to communicable and non-communicable diseases. Regarding our original three research questions, we respond in turn. First, how do LMICs cluster together across labour market regulations, measured with inequality and poverty indicators? Based on labour market inequality and poverty indicators, LMICs clustered into six labour market groups: Residual, Emerging, Informal, Post-Communist, Less Successful Informal, and Insecure. Second, what is the strength of association between labour market regulations and population health? Labour market inequality and poverty and population health were strongly correlated in low-income countries, but only labour market poverty and health was significant in middle-income nations. Improving material living conditions in LMICs are crucial to enhancing population health through strengthening labour market regulations (e.g., decreasing levels of child labour and poor workers). Third, are more egalitarian labour market clusters associated with better population health outcomes? More egalitarian clusters exhibited better health outcomes compared to their cluster counterparts and health distributions were graded across labour market clusters.

Given the dearth of research on labour markets and population health, this study's most key contribution is the development of labour market taxonomies in LMICs. Middle-income clusters consisted of relatively advanced industrialized East Asian and Eastern European countries, less industrialized countries of Latin America, and marginally industrialized countries. Noticeably, East Asian countries and East European countries clustered together to form the Residual group. While the former failed to develop or implement strong labour market regulations and encouraged instead the private sector to meet the citizenry's welfare needs [56], the latter have succeeded toward strengthening labour markets through redistributive and conservative welfare systems [15,50]. Common to all Residual countries includes the extent of growing industrialization, and more importantly, the incorporation of rural farmers into the urban working class [51].

Emerging labour markets, or Cluster 2 of middle-low countries, represents the Latin America's upward industrialization (e.g., import substitution type of developmental strategy)[51,57]. Emerging markets offer an interesting contrast to the experience of East Asian nations. On one hand, East Asian "tigers" are ideologically important to the US because of their proximity to the former Soviet Union border. In contrast, Latin America's political environment is comparably more independent to advance the interests of workers and buffers the effects of imperialism. This has contributed to the rise of center-left and left-leaning governments in Latin America that often cultivate their support from urban formal sector workers, who in return, enjoy relatively more generous welfare benefits from governments compared to East Asian countries [51,58,59].

The Informal cluster of middle-income countries represents a mixture of three different types of countries: first are Middle Eastern countries such as Bahrain, Oman, and Saudi Arabia, which predominantly rely on export of petroleum as their primary economic activity. The second are industrialized countries in Africa such as Botswana and Tunisia. El Salvador, Belize, and Turkey form a third sub-cluster, relying primarily on tourism and agriculture for economic growth. Despite variations in GDP per capita and geographical location, Informal labour markets share common industrial specializations, which limit the ability of workers to organize and increase the availability of informal contracts.

Low-income countries represent another level of labour market instability altogether. These countries are similarly impoverished yet critical variations exist. For example, Cluster 1 within low-income countries consists of post-communist republics that seceded from former countries with vestigial forms of welfare states. Failed African states and other similarly unstable countries represent the Insecure cluster where labour contracts are notoriously difficult to enforce [36] and health indicators are predictably worse. Poor levels of population health are primarily attributed to general economic and political disequilibrium [36], rather than to the character of labour market conditions or regulations.

This study's analytic methods and findings largely complement other comparative research on global regimes, labour markets, working conditions, and welfare outcomes. Specifically, our work builds on Gough and Wood's comparative welfare regimes framework [36,38] and Rosskam's research using the Work Security Index (WSI) [60]. Guided by Gough and Wood's distinction between informal security and security regimes, we used national income and labour market characteristics to distinguish transitional (e.g., middle-income) and developing (e.g., low-income) countries and to investigate cross-national distributions of population health. By shifting the research focus from developed contexts to developing ones, we advance the analysis of labour market institutions to include informal regulations as important determinants. Our mapping of labour market clusters largely mirror Gough and Wood's cluster analysis of welfare regimes. For example, Gough and Woods's "Actual or Potential Welfare State Regimes" represents their most advantaged cluster (e.g., Thailand, several Eastern European and Latin American countries) because these countries are characterized with high state commitments and high welfare outcomes. Our most advantaged labour market cluster, Residual, includes these same countries and consistently ranked as the healthiest cluster. At the other extreme, our Insecure cluster resembles Gough and Wood's "Externally Dependent Insecurity Regime" given that both consist of sub-Saharan Africa countries with predatory forms of capitalism, high dependencies on foreign aid, and very poor welfare and health outcomes.

This study also augments Rosskam's recent work using the WSI, which was developed by the ILO's Socio-Economic Security Programme [61] as a benchmarking system to compare industrialized and industrializing countries on the extent governments protect working populations' health, safety, and well-being [62,63]. Findings using the WSI cross-validate our methods and results in two important ways. First, Rosskam [60] found that women workers are most disadvantaged with respect o social and economic insecurities and inequalities. This finding substantiates the gendered dimensions of work and provides support to our use of 'estimated earned income ratio between male and female workers' and 'labour force participation gap between female and male workers' to construct our labour market inequality factor score. Second, the most critical cases of worker insecurities are found in the most economically deprived countries in Africa (e.g., Guinea-Bissau, Mauritania, Rwanda), Asia (e.g., Indonesia, Nepal, China, India) and Eastern Europe (e.g., Albania, Armenia, Bulgaria) [60] p. 276. Figures 1, 2, 3, and 4 confirm this finding, in that, the worst population health distributions are found in low-income countries, and in Post-Communist (e.g., Albania, Armenia, Bulgaria, China, Indonesia), Less Successful Informal (e.g., India, Mauritania), and Insecure (e.g., Guinea-Bissau, Nepal, Rwanda) clusters.

### Study limitations

Given the exploratory nature of our study, several limitations warrant further attention. First, our interpretation adopts a "top-down", macro approach to understanding the impact of labour markets on population health rather than a "bottom-up", micro approach, which represents the more common method within social and health policy literatures. We acknowledge that "bottom-up" effects such as community or labour organizing has the potential to influence macro structural changes (e.g., increasing worker's bargaining power, voting for pro-labour political parties). Though the social mechanisms responsible for population health are non-recursive and reciprocal, we did not test alternative pathways. Second, an alleged weakness of taxonomy construction is its lack of predictive power. To assess the usefulness our taxonomy, we compared our labour market clusters against Gough and Wood's [36] global welfare regimes and found high agreement between both classifications (e.g., informal security regimes mapped onto middle-income countries and insecurity regimes mirrored low-income nations). Cluster techniques have been criticized for its macro-level focus at the expense of overlooking inequalities within-countries [64]; however, we counter that identifying labour market clusters remains instructive to bringing to light the political and economic contexts of global health [37]. Third, our data represents a limited time period from 2000 to 2004. This is potentially problematic because the health impact of labour market policies and regulations is time-dependent. Our results should be interpreted as heuristic and as a proxy for long-term labour market effects. Future studies should take advantage of time-series data (e.g., measurements equally spaced through time) and methods (e.g., time-domain, frequency domain) to make valid inferences on the health impact of labour markets over time [65]. Forth, our 6 labour market clusters shows much heterogeneity, resulting in part from the limitations associated with quantitative and macro-comparative approaches. Some countries do not entirely conform to the explanations provided for a given cluster (e.g., the Philippines and El Salvador are not Post Communist or oil rich countries, respectively). Since our empiricist approach reduces data at the expense of finer distinctions, future work should elaborate on country clusters using methods that can account for historical, political, and economic factors.

## Conclusions

This study's findings support our thesis that labour market regulations, expressed as levels of inequality and poverty, are important social determinants of population health among LMICs. Labour market regulations can affect workers' health along two different pathways. The first relates to the physical and psychosocial conditions of work, which has been traditionally the focus of occupational health [66,67]. The second is the economic outcome of the labour process, usually expressed in wages and benefits [24]. Workplace hazards and the economic compensation affect workers' health via several mechanisms [3].

Our categorisation of LMICs into labour market clusters reveals two important distinctions. First, it highlights the consequential difference between middle-income (informal security regimes) and low-income (insecurity regimes) countries. The labour markets of middle-income countries are simultaneously characterized by increasing levels of stability and emerging labour market institutions, which provide the necessary institutional landscape to generate labour movements and strengthen welfare systems (e.g., Chile) [58]. Second, the economies of low-income countries are hindered by a heavy reliance on informal work. Large informal sectors tend to result in severe labour market insecurities. Aggravating these insecurities among low-income countries are extensive conflicts, authoritarian regimes, and foreign interventions that undermine the rule of law and the protection of workers [36,68]. As demonstrated, this study makes an original contribution to global health scholarship, provides direction for further research, and demonstrates that labour markets can be systematically investigated with a combination of labour market and welfare regime approaches.

## Abbreviations

(EPL-Index): Employment protection legislation index; (ILO) International Labour Organization; (LMICs): Low- and middle-income countries; (GNPpc): Gross National Product per capita; (HALE): Health adjusted life expectancy; (KILM): Key Indicators Of Labour Markets; (PCA): Principal component analysis; (SDOH): Social determinants of health; (OECD): Organization for Economic Co-Operation and Development; (WHOSIS): World Health Organization's Statistical Information System; (WSI): Work Security Index.

## Endnotes

^1 ^Health outcomes are presented as box plots, which represent the middle 50% of the data, ranging from the upper boundary (75th percentile) to the lower boundary (25th percentile). Box lines indicate median values. Vertical lines extending from the box indicate minimum and maximum values and dots are outliers.

## Competing interests

The authors declare that they have no competing interests.

## Authors' contributions

CM and HC conceived and designed the study and acquired the data. All authors analyzed and interpreted data, drafted and revised the manuscript, and approved the final version. All authors read and approved the final manuscript.

## Pre-publication history

The pre-publication history for this paper can be accessed here:

http://www.biomedcentral.com/1471-2458/12/286/prepub
